# Humanized Mouse Models of Rheumatoid Arthritis for Studies on Immunopathogenesis and Preclinical Testing of Cell-Based Therapies

**DOI:** 10.3389/fimmu.2019.00203

**Published:** 2019-02-19

**Authors:** Katina Schinnerling, Carlos Rosas, Lilian Soto, Ranjeny Thomas, Juan Carlos Aguillón

**Affiliations:** ^1^Programa Disciplinario de Inmunología, Immune Regulation and Tolerance Research Group, Facultad de Medicina, Instituto de Ciencias Biomédicas, Universidad de Chile, Santiago, Chile; ^2^Departamento de Ciencias Biológicas, Facultad de Ciencias de la Vida, Universidad Andrés Bello, Santiago, Chile; ^3^Departamento de Ciencias Morfológicas, Facultad de Medicina y Ciencia, Universidad San Sebastián, Santiago, Chile; ^4^Unidad de Dolor, Departamento de Medicina, Hospital Clínico Universidad de Chile, Santiago, Chile; ^5^Diamantina Institute, Translational Research Institute, Princess Alexandra Hospital, University of Queensland, Brisbane, QLD, Australia

**Keywords:** rheumatoid arthritis, humanized mice, transgenic mice, mouse/human chimera, preclinical model, cell-based immunotherapy

## Abstract

Rodent models of rheumatoid arthritis (RA) have been used over decades to study the immunopathogenesis of the disease and to explore intervention strategies. Nevertheless, mouse models of RA reach their limit when it comes to testing of new therapeutic approaches such as cell-based therapies. Differences between the human and the murine immune system make it difficult to draw reliable conclusions about the success of immunotherapies. To overcome this issue, humanized mouse models have been established that mimic components of the human immune system in mice. Two main strategies have been pursued for humanization: the introduction of human transgenes such as human leukocyte antigen molecules or specific T cell receptors, and the generation of mouse/human chimera by transferring human cells or tissues into immunodeficient mice. Recently, both approaches have been combined to achieve more sophisticated humanized models of autoimmune diseases. This review discusses limitations of conventional mouse models of RA-like disease and provides a closer look into studies in humanized mice exploring their usefulness and necessity as preclinical models for testing of cell-based therapies in autoimmune diseases such as RA.

## Introduction

Rheumatoid arthritis (RA) is a chronic inflammatory disorder which affects the synovial tissue of the joints, causing articular pain and disability (1). Initially manifested locally, RA later develops into a systemic disease that involves major organ systems and reduces life expectancy (2). The disease is characterized by an infiltration of the synovium with inflammatory cells, proliferation of synovial fibroblasts, forming an invasive pannus that destroys the adjacent cartilage and bone, and progressive joint damage (3).

Rodent models have been used over decades to study the immunopathogenesis of RA and to test the efficacy of anti-rheumatic drugs (4). There are numerous rodent models of RA-like disease, each mirroring certain aspects of the disease (4–6). Important findings have emerged from studies using these models, such as the need for CD4+ T cells and B cells for the development of RA (7, 8), the importance of pro-inflammatory cytokines in RA pathogenesis (9–11), and the discovery of RA-relevant autoantigens (12, 13). Nevertheless, mouse models of RA reach their limit when it comes to testing of new therapeutic approaches such as cell-based therapies. More than 80% of potential therapeutics, which have been shown to be safe and effective in animal studies, fail when tested in humans (14, 15). One example is the lack of therapeutic efficacy of interleukin (IL-) 17 inhibitors in RA patients (16, 17), although suppression of IL-17 signaling had been shown to reduce joint inflammation as well as cartilage and bone destruction in mice (18). Similarly, inhibition of IL-1 signaling had been demonstrated to ameliorate arthritis in mice (19), while anti-IL-1 therapy displayed limited efficacy in RA patients (4, 20). Differences between the human and the murine immune system make it difficult to transfer the results from the mouse model to patients (21, 22). Consequently, translational research needs to refocus on human, and not mouse, immunology (23, 24). This is particularly important for preclinical testing of therapeutic approaches based on dendritic cells (DCs). While monocyte-derived DCs are used for clinical applications in humans (25), DCs obtained from bone marrow precursors are administered in mouse models (26, 27), which considerably affects the transferability of the results from mice to men.

**Figure 1:**
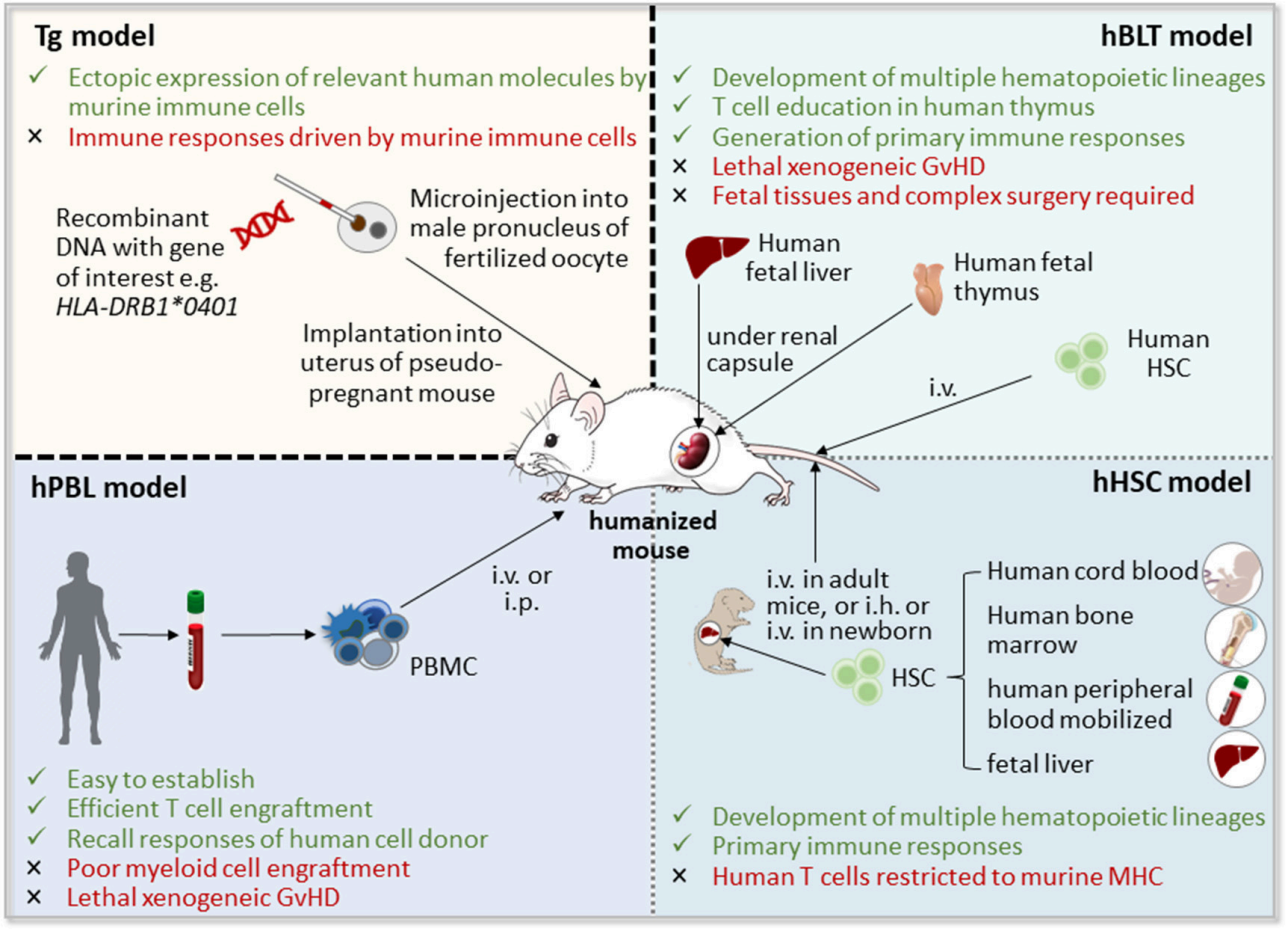
Strategies for the generation of humanized mice [adapted from Shultz (30) and Hahn et al. (31)]. Humanized mice can be obtained by the introduction of human transgenes, such as human leucocyte antigen (HLA) class II molecules, which will then be expressed by mouse immune cells, or by the generation of mouse/human chimera through implantation of human cells, including hematopoietic stem and progenitor cells (HSCs) and peripheral blood mononuclear cells (PBMCs), and/or human tissues, such as fetal liver and thymus, into immunodeficient mice. GvHD, graft vs. host disease; hBLT, human bone marrow-liver-thymus-engrafted; hPBL, human peripheral blood lymphocyte-engrafted; i.h., intrahepatic; i.p., intraperitoneal; i.v., intravenous; Tg, transgenic mice.

Since studies of human immune responses and disease *in vivo* are limited by ethical and technical constraints, there is a need for animal models that on the one hand accurately mirror the pathogenesis of the autoimmune disease, and on the other allow pre-clinical testing of cell-based therapeutic approaches targeting human cells and tissues *in vivo*. To tackle these issues, humanized mice have been developed using two different strategies: (i) the introduction of human RA-relevant transgenes such as human leukocyte antigen (HLA) molecules, T cell receptors (TCR), or autoantigens (28), and (ii) the generation of mouse/human chimera by the engraftment of human (RA-derived) cells and/or tissues into immunodeficient mice (29) (Figure 1). Combination of both approaches will lead to more sophisticated humanized models of autoimmune diseases such as RA (32).

This review provides an overview of mouse models of RA-like disease and their limitations and discusses different humanization strategies for the generation of RA models, with focus on the use of humanized mice as tools for pre-clinical testing.

## Immunopathogenesis of Rheumatoid Arthritis and the Prospects of Cell-Based Therapy

Although the etiology of RA is not fully known, its autoimmune nature has been widely recognized. An interplay between genetic predisposition and environmental factors, such as smoking and microbial infections, are thought to trigger the development of the disease (33). The contribution of genetic factors to RA pathogenesis is mainly attributed to certain HLA alleles of the major histocompatibility complex (MHC) class II (34). Besides presenting antigen peptides to CD4+ T cells in the periphery, HLA class II molecules are also responsible for the selection of the CD4+ TCR repertoire in the thymus and thus control the release of autoreactive cells (35, 36). A consensus amino acid motif in the P4 peptide-binding pocket of the β1 subunit of the HLA-DR molecule, denoted “shared epitope” (SE), is a major risk factor of RA (37, 38). Among HLA-DR alleles containing the SE, DRB1^*^0401, and ^*^0101 have been described most extensively in the context of RA (39, 40). Various peptides derived from endogenous joint proteins, such as type II collagen (CII), cartilage proteoglycan aggrecan, and human cartilage glycoprotein (HCgp)-39, have been shown to bind to SE-containing HLA-DR molecules and to be specifically recognized by T lymphocytes from RA patients (13, 41–44). Particularly, peptides post-translationally modified by citrullination bind with high affinity to the SE, initiating citrulline-specific T and B cell responses (45, 46). Citrullination is catalyzed by peptidylarginine deiminases (PAD), which convert the amino acid arginine into citrulline, leading to a loss of positive charge that might render self-peptides immunogenic (47). Several citrullinated peptides, including fibrinogen, vimentin, α-enolase, aggrecan, and CII are present in RA joints and are targets of lymphocyte responses in RA patients carrying the SE (48, 49). Antibodies directed against citrullinated proteins/peptides (ACPAs) are specific to RA and associated with the presence of the SE and with increased disease severity (50, 51).

Antigen-presenting cells, particularly DCs, are key players in the initiation and maturation of the autoimmune response in RA (52). DCs can activate self-reactive CD4+ T cells by presenting autoantigens in the context of MHC class II molecules and providing costimulatory and pro-inflammatory signals (53–55). Activated autoreactive CD4+ T cells differentiate into inflammatory T helper (Th) cell subsets producing either interferon (IFN)-γ and tumor necrosis factor (TNF) (Th1), IL-17 and IL-21 (Th17), or a mixed cytokine profile (Th1/17), and accumulate in the inflamed joint (56–58). These autoreactive T cells drive the differentiation of B lymphocytes into plasma cells producing autoantibodies such as ACPAs (59), which in turn promote osteoclast differentiation and activation, leading to cartilage and bone erosion (33). Autoreactive CD4+ T cells also stimulate macrophages and synovial fibroblasts to secrete pro-inflammatory mediators, including TNF, IL-1, and IL-6, which contribute to synovial inflammation through recruitment of immune cells and expansion of synovial fibroblasts, forming an invasive pannus (60, 61).

Current treatment options of RA involve non-steroidal anti-inflammatory drugs, glucocorticoids and disease-modifying anti-rheumatic drugs, either conventional, such as methotrexate, targeted, like Janus kinase inhibitors, or biologics, including antagonists of proinflammatory cytokines, modifiers of T cell co-stimulation and B cell-depleting antibodies (1, 62). Besides the fact that a proportion of patients remain refractory to treatment (63), none of these drugs provides a cure of RA, requiring life-long treatment, associated with a progressive loss of efficacy, toxicity, and the appearance of serious adverse effects (64–66). A promising strategy to restore self-tolerance and thereby achieve long-term remission, is the depletion or reprogramming of autoreactive T cells by regulatory T (Treg) cells or tolerogenic DCs (tolDCs) (25, 67, 68). Naturally occurring CD4+ Treg cells are characterized by the constitutive expression of the transcription factor FOXP3 and IL-2 receptor α-chain (CD25). Treg maintain immunological tolerance and prevent autoimmune diseases by suppressing the activation and proliferation of self-reactive effector T cells (67). Although Treg cells are present in the synovial fluid of RA patients, these Treg cells fail to inhibit Th1 responses (69, 70). Strategies targeting Treg cells for the treatment of RA include (i) the expansion of autoantigen-specific naturally occurring Treg cells *in vivo*, (ii) their propagation *in vitro* and subsequent transfer back into the host, and (iii) the conversion of antigen-specific T cells into Treg cells *in vitro* or (iv) *in vivo* (67). Dendritic cells (DCs) are professional antigen-presenting cells that instruct T cells, according to the surrounding environment, to mediate immune responses or tolerance. TolDCs with immunoregulatory properties can be generated *in vitro* from monocytes or hematopoietic stem cells and are able to control aberrant CD4+ T cell responses through the induction of anergy, conversion of T effector into Treg cells, or deletion of autoreactive T cells (71–74). An important advantage of tolDC- or Treg-based therapy over conventional treatment of RA is its potential to modulate immune responses in an antigen-specific manner, which might permit a selective downregulation of autoreactive lymphocyte responses while avoiding a general shutdown of immunity against pathogens. Both Treg cell and tolDC-based approaches have been extensively tested in conventional mouse models of RA-like disease (75) and the safety of tolDCs has even been approved in phase I/II clinical trials (76, 77). Nevertheless, sophisticated mouse models that accurately recapitulate human RA are still missing. Humanized mouse models of RA might help to predict the efficacy and side effects of cell-based approaches in further clinical trials, as well as to adjust parameters, such as dose, injection route, and required dosing interval.

## Conventional Mouse Models of Rheumatoid Arthritis and Their Limitations

Numerous rodent models of RA are available, each of which mirrors particular aspects of the disease (4, 6). These conventional models represent classic hallmarks of RA, such as joint swelling, synovitis, pannus formation, and bone erosion, but differ in the mechanisms of induction and launched immune processes, as well as in their speed of onset, chronicity, and severity (6, 78). A distinction is made between induced and spontaneous models. In induced models, non-specific immune activation, cartilage-directed autoimmunity, or abundant exogeneous/infectious triggers cause RA-like disease, while in spontaneous models, arthritis develops without deliberate immunization and is non-limiting, providing a chronic situation like in human RA (5, 79, 80). The most frequently used models are introduced below.

### Induced Rodent Models of RA-like Disease

Adjuvant arthritis (AA) was the first described animal model of RA and can be induced by a single intradermal injection of complete Freund's adjuvant (CFA), containing heat-inactivated mycobacteria, at the base of the tail in Lewis rats (81) or by repetitive intra-articular CFA injection in DBA/1 or C57BL/6 mice (82). The hallmark of AA is its rapid onset and progression to polyarticular inflammation, leading to a chronic erosive disease with severe joint malformation (6). The disease is driven by CD4+ T cells (83) and susceptibility to develop AA is related to MHC and non-MHC genes (84). Originally, it was assumed that mycobacterial components, such as 65k heat shock protein, cross-react with self-antigens from joint cartilage in this model (85). However, it has been shown that nonimmunogenic adjuvants such as avridine, muramyl dipeptide, pristane, and incomplete Freund's adjuvant also induce AA in many rat strains and mice, indicating that adjuvants may enhance autoreactivity to articular antigens (83, 86–88). Unlike in human RA, the AA model displays not only bone erosion, but also bone apposition at early stages of the disease with limited to no cartilage damage (79).

Collagen-induced arthritis (CIA) is the most commonly used model of RA-like disease (89). In this model, severe joint inflammation is induced through immunization with CII, a major component of hyaline cartilage, together with CFA (6, 90). Susceptibility to CIA is related to the murine MHC class II molecule H-2^q^ whose peptide-binding pocket has a similar primary structure like the SE of RA-associated HLA-DR molecules (91, 92). Although several mouse strains are susceptible to CIA, the DBA/1 strain is the “gold standard” of this model (90). Autoreactive CD4+ T cells are required for the induction of CIA (7, 93, 94), synovial proteins are subjected to PAD-induced citrullination and an association of anti-CII antibodies and ACPA to the development of arthritis has been described (95, 96). The passive transfer of polyclonal immunoglobulin (Ig) G from sera of CIA mice or monoclonal anti-CII antibodies induces arthritis even in mouse strains that are not susceptible to CIA, indicating an important role of autoantibodies in the effector phase of the disease (97, 98). Important limitations are that CIA is an acute model, in which remission occurs at 10–14 days after disease onset (99), extra-articular manifestations are due to CFA (100) and disease severity is highly variable and dependent on environmental factors, such as grouping stress (101). Joint inflammation in CIA mice is particularly mediated by Th17 cells (102), while the pathogenesis of human RA involves Th1, Th17, and Th1/17 cells (56–58).

Proteoglycan-induced arthritis (PGIA) is induced by immunization with human cartilage proteoglycan aggrecan in susceptible BALB/c or C3H mice strains (12). The progressive chronic polyarthritis in the PGIA model shares features with human RA such as deposition of immune complexes in the joint and the presence of rheumatoid factor autoantibodies (6, 12). Susceptibility to PGIA in BALB/c mice is associated with the presentation of the dominant epitope aggrecan 89–103 by I-A^d^ MHC class II molecules (103). PGIA development is dependent on B cells, which present antigen and produce anti-PG antibodies, as well as on CD4+ T cells, which provide help to B cells for antibody production. Antibodies are cross-reactive between human proteoglycan, used for immunization, and self (mouse) cartilage proteoglycan (104, 105). The choice of adjuvant and route of immunization determine the prevalent Th subset in the PGIA model. Immunization with CFA and subcutaneous injection route result in predominant Th17 responses, whereas dimethyldioctadecyl-ammonium bromide (DDA) as adjuvant and intraperitoneal injection induce more Th1 cells (106, 107).

Antigen-induced arthritis (AIA) is triggered by the injection of exogeneous antigen, such as methylated bovine serum albumin (mBSA), into the joint (108). This robust model of destructive bystander arthritis develops almost independently of the genetic background of mice and leads to a moderate and limiting local disease involving both T cells and immune complexes (79, 109).

RA-like disease can also be passively transferred to naïve mice by injecting serum antibodies against endogenous CII, PG, or glucose-6-phosphate isomerase (GPI) (79, 98, 110, 111). These immune complex arthritis models are almost independent of the genetic background of mice and resemble the effector phase of the disease, in which excessive immune complex formation at joint tissues triggers complement activation and the release of inflammatory mediators through Fcγ receptor engagement on phagocytes, leading to rapid onset, but transient destructive arthritis (78). Administration of pathogenic immunoglobulins is sufficient to confer disease and does not require T or B lymphocytes, nevertheless, T cells have an enhancing effect on autoantibody-induced arthritis (112).

### Spontaneous Mouse Models of RA-like Disease

Spontaneous models include genetically modified mice, such as SKG, K/BxN, human TNF transgenic (Tg) and IL1ra^−/−^ mice (5, 80). In SKG mice, arthritis development is attributed to a missense mutation in the TCR signaling adaptor molecule ZAP70, leading to a defective negative selection in the thymus and the release of autoreactive T cells (113). SKG mice establish arthritis following stimulation of the innate immune system with zymosan or other microbial triggers, while housing under pathogen-free conditions or treatment with antibiotics completely block arthritis development (114, 115).

The K/BxN model was discovered coincidentally by crossing KRN mice, which express an transgenic TCR, specific for an epitope of bovine pancreas ribonuclease, with autoimmune-prone non-obese diabetic (NOD) mice. The offspring, referred to as K/BxN, spontaneously developed severe arthritis (116), driven by the activation of T cells expressing the KRN-derived TCR, which, due to cross-reactivity, recognize the ubiquitously expressed self-protein GPI bound to the NOD-derived MHC class II molecule I-A^g7^ (117, 118). Activated autoreactive T cells promote polyclonal B cell activation and T helper cell-dependent production of GPI-specific IgG autoantibodies (110, 119). Transfer of serum IgG from arthritic K/BxN mice has been shown to induce robust and reproducible arthritis in healthy mice, indicating that autoantibodies might directly trigger joint inflammation (110, 120). However, it must be considered that GPI might not be an essential or RA-specific autoantigen.

TNF transgenic (Tg) mice, which constitutively express human TNF, spontaneously develop inflammatory, highly erosive polyarthritis, similar to human RA, which can be completely prevented by treatment with monoclonal antibodies against human TNF (121). When these TNF-overproducing mice were crossed to a severe combined immunodeficiency (SCID) background, lacking mature B and T cells, inflammatory arthritis still develops, indicating that TNF acts downstream of lymphocyte responses in this model (122). Thus, TNF Tg mice are not suitable for testing of therapeutic approaches that target lymphocyte responses in RA.

Mice overexpressing human IL-1α Tg present chronic destructive polyarthritis, characterized by hyperplasia of the synovial lining, pannus formation and cartilage destruction (123). However, pathogenesis of arthritis in hIL-1α Tg mice is mediated by monocytes/macrophages and activated neutrophils, while T and B lymphocytes are sparse (123). In an opposite approach, deletion of IL-1 receptor antagonist (IL-1ra), an endogenous inhibitor of IL-1 signaling, leads to a spontaneous arthritis model in which T cells are main actors (10, 124). Increased levels of antibodies against IgG (rheumatoid factor), CII and double-stranded DNA also point to the development of an autoimmune response in IL1ra^−/−^ mice (124).

### Achievements and Obstacles of Testing Cell-Based Strategies in Conventional Mouse Models of Rheumatoid Arthritis

Cell-based therapeutic approaches have mainly been tested in induced models of RA-like disease (75). Adoptive transfer of antigen-specific Treg, either expanded from natural occurring Treg, or induced from conventional T cells, e.g., by enforcing the expression of the Treg-specific transcription factor FoxP3 or reprogramming through tolDCs, has been shown to suppress the progression of arthritis in models of CIA, PGIA and AIA (125–129). Therapeutic effects of tolDCs, differentiated *in vitro* from precursors and genetically modified to express IL-4, or modulated by either short stimulation with bacterial lipopolysaccharide (LPS), vasoactive intestinal peptide, a combination of dexamethasone, vitamin D3 and MPLA, or the NF-κB inhibitor Bay11-7082, have been reported in mouse models with established CIA and AIA (26, 27, 130–132). However, testing of tolDC-based therapeutic strategies in conventional mouse models is limited by the fact that, due to technical constraints, administered murine DCs are generated from bone marrow progenitors, while monocyte-derived DCs are used for clinical applications in humans (25). It is evident that bone marrow- and monocyte-derived DCs represent different DC subsets which might have an altered or even opposite effect on the disease *in vivo*.

A common weakness of all conventional models of RA is that inflammatory responses are completely mediated by the murine immune system, which differs in its components and organization from the human immune system. Discrepancies in innate and adaptive immune mechanisms between both species include the composition of leukocyte subsets in peripheral blood, Toll-like receptors, Fc receptors, Ig subclasses, B cell and T cell signaling pathways, γδ T cells, response to IFN-γ, cytokines and cytokine receptors, expression of costimulatory and adhesion molecules, chemokine and chemokine receptor expression, as well as expression of receptors involved in the uptake of phagocytic cargo, production of reactive oxygen species and IL-1 by monocyte subsets, among others (21, 22, 133). While Foxp3 defines the Treg population in mice, human FOXP3+ T cells are heterogeneous in their function (67). In response to stimulation, human CD4+ effector T cells express MHC class II molecules (134) and upregulate FOXP3 without acquiring immunoregulatory functions (135), while mouse T cells do not. In CIA mice, γδ T cells are the predominant source of IL-17 in affected joints, but they are nearly absent in joints of RA patients, where Th1 cells dominate (136). Another difference between conventional animal models of RA-like disease and human RA is their lack of gender bias (137).

Due to these species-specific differences, it is not surprising that several promising therapeutic principles found in mouse models do not work in humans (15, 16, 20). This hampers the translation of experimental data obtained from conventional murine models of RA-like disease to clinical applications in patients and creates a demand for humanized models which accurately mimic the human disease (Table 1).

**Table 1:** Comparison between conventional and humanized mouse models of rheumatoid arthritis.

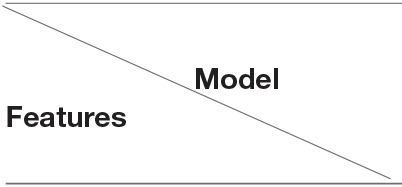	**Conventional mouse models of RA**	**Humanized mouse models of RA**	
		**Transgenic mice**	**Mouse/human chimera**
Autoimmune response-driving cells	Mouse immune cells	Mouse immune cells (with ectopic expression of human Tg)	Human T and B cells, mouse macrophages and granulocytes
Relevant MHC molecules	Mouse MHC (CIA: H-2^q^; PGIA: I-A^d^; K/BxN: I-A^g7^)	Human MHC/HLA class II (DR1, DR4 or DQ8)	HLA alleles of the human cell donor
Involved antigen	Articular, non-articular or exogeneous antigen	Restriction to already known antigen epitopes	Multiple synovial antigens
Autoantibodies	Only in induced models and K/BxN mice	Yes	Yes (except HSC-engrafted mice)
Disease development	Induced or spontaneous	Induced by immunization with antigen (and adjuvant)	Spontaneous (except HSC-engrafted mice)
Disease incidence	Moderate to high	Variable (14–100%), depending on the genetic background of mice	Variable, depending on the type of the human graft and disease status of the donor
Disease onset	Rapid (induced models, K/BxN) or slow (SKG, IL1ra^−/−^)	Rapid	Rapid
Disease severity	Moderate to severe	Severe	Dependent on the human donor
Disease duration	Self-limiting (induced models, except PGIA) or chronic (spontaneous models)	Self-limiting or chronic	Limited by onset of GvHD
Gender bias	No (except CIA: male bias, in contrast to human RA)	Yes, more frequent in female mice	Yes, corresponding to human cell donor
Dependence on strain	Yes (except AIA, TNF Tg, IL1ra^−/−^)	No	Yes, immunodeficient strains lacking T, B, and NK cells
Value as pre-clinical model for testing cell-based therapies	Testing of approaches based on the murine cell-equivalent with limited predictive value for clinical application	Testing of approaches based on murine cells expressing relevant human MHC class II or TCR; Restricted to well-defined antigen epitopes	Testing of human cell-based therapies in a human cell environment within mice; closest approximation to clinical application in patients

*AIA, antigen-induced arthritis; CIA, collagen-induced arthritis; MHC, major histocompatibility complex; GvHD, graft vs. host disease; HLA, human leukocyte antigen; HSCs, hematopoietic stem and progenitor cells; PGIA, proteoglycan-induced arthritis; TCR, T cell receptor*.

## Transgenic Mice as Humanized Models of Rheumatoid Arthritis

**Figure 2:**
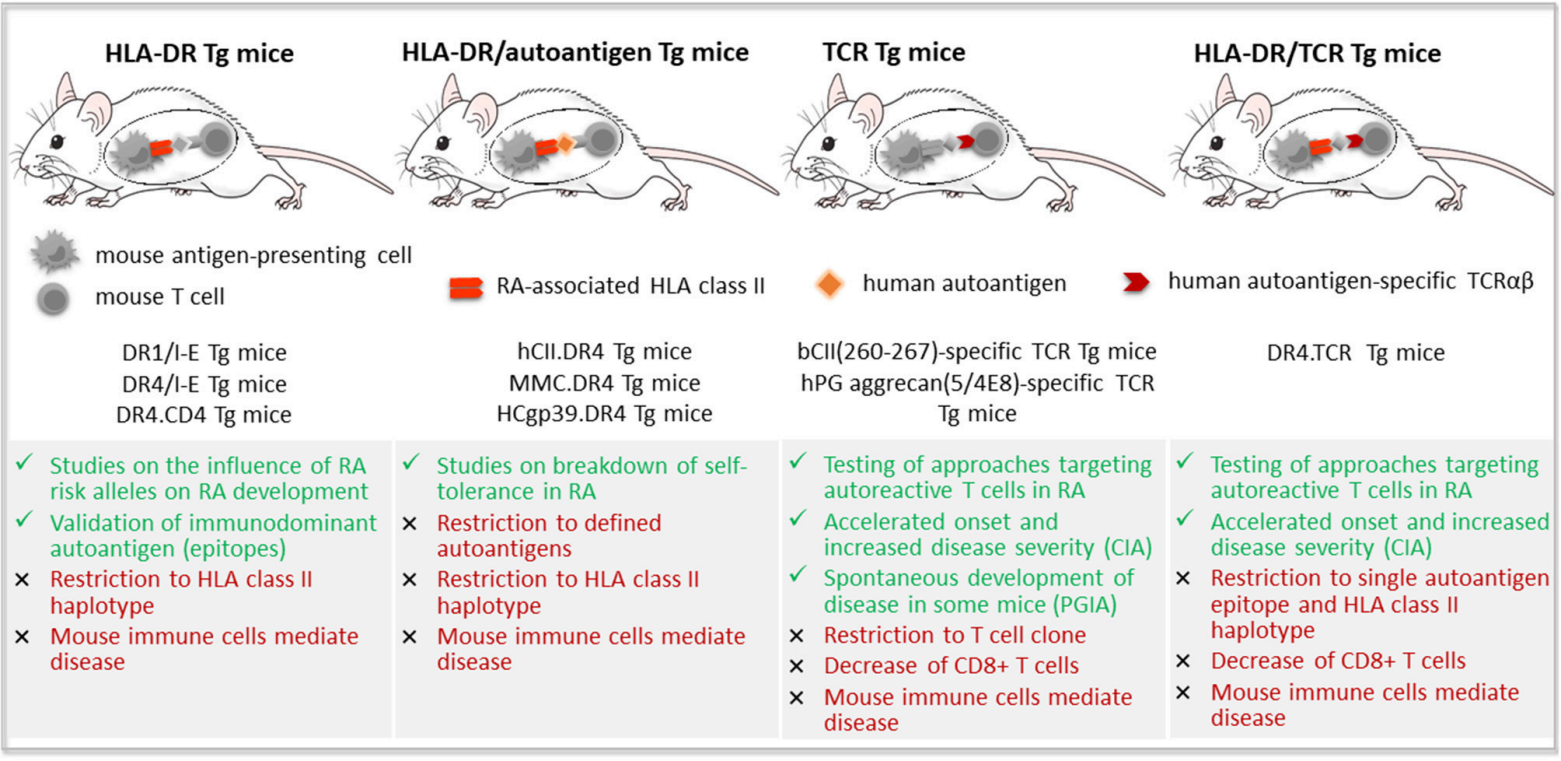
Transgenic mouse models of rheumatoid arthritis. Transgenic (Tg) mouse models of rheumatoid arthritis (RA) reported in the literature include mice expressing (i) RA-susceptible human leukocyte antigen (HLA) class II molecules, such as DRB1*0101 (DR1) or DRB1*0401 (DR4); (ii) human RA-associated autoantigens, such as type II collagen (CII), either complete or as mutated mouse collagen (MMC) containing the immunodominant epitope, and human cartilage glycoprotein-39 (HCgp-39); (iii) an autoantigen-specific T cell receptor (TCR) alone; or (iv) together with RA-susceptible HLA-DR molecules. Advantages are depicted in green color, limitations in red.

This humanization strategy is based on transgenic expression of human molecules, such as HLA class II, RA-associated synovial autoantigens and/or an autoantigen-specific T cell receptor, in immunocompetent mice (Figure 2).

### Human Leukocyte Antigen Class II Transgenic Mouse Models

The advent of mice that lack endogenous MHC class II molecules and express human HLA transgenes instead has significantly advanced the understanding of the importance of certain HLA class II alleles, particular those containing the SE, in the development of RA (137). The basis for HLA class II transgenic mice was created in 1991, with the generation of a mouse with disrupted expression of the murine MHC class II genes I-A and I-E, lacking mature CD4+ T cells (138). First attempts to introduce human HLA class II transgenes in these murine MHC class II-deficient mice resulted in low CD4+ T cell selection, due to poor interaction between mouse CD4 and human HLA class II molecules (139). To circumvent this problem, two strategies have been pursued: generation of double transgenic mice expressing the human HLA gene in all antigen-presenting cells and human CD4 in all T cells (140–142), or introduction of a chimeric HLA/I-E molecule composed of the peptide-binding domain from human DR and the CD4-binding domain derived from mouse I-E (143, 144). This way, the HLA class II transgene was able to positively select CD4+ T cells expressing diverse Vβ TCRs, resulting in normal development of the CD4+ T cell compartment and maintenance of peripheral tolerance to transgenic HLA molecules (137).

The first strategy was used by Fugger and colleagues, generating HLA-DRA1^*^0101/-DRB1^*^0401 (DR4)/human CD4 Tg mice which did not develop spontaneous autoimmune diseases, but were susceptible to CIA (140, 142). Rosloniec and coworkers followed the second approach to establish Tg mice expressing chimeric HLA-DRB1^*^0101 (DR1)/I-E or DR4/I-E, which developed severe autoimmune arthritis following immunization with bovine or human CII, accompanied by strong DR1 and DR4-restricted T and B cell responses, respectively (13, 145). The incidence of CIA varied from 14 to 100%, according to the introduced human HLA-DR transgene, the genetic background of mice, and the applied immunization protocol (13, 142, 146). In both DR1 Tg and DR4 Tg mice, T cell response was focused to position 259–273 of human CII, suggesting that different RA-associated HLA-DR molecules containing the SE bind and present the same immunodominant epitope of CII (13, 145). Even though both HLA-DRB1^*^0401 (RA-susceptible) and ^*^0402 (RA-resistant) molecules present CII and its derived peptide, only ^*^0401 Tg mice develop a pro-inflammatory response (146). Furthermore, comparison of T cell polarization and immune responses between ^*^0401 and ^*^0402 Tg mice encouraged the hypothesis that the SE selects T cells with a predetermined Th17-biased cytokine profile that efficiently clear infections, but may, on the other hand, drive autoimmunity in response to pathogens or certain environmental factors (147). Interestingly, Tg mice carrying an RA-susceptible haplotype mimic the gender-bias of RA, displaying an increased susceptibility to develop the disease as well as a stronger cellular and humoral response to CII in females (146).

To date, DR4 Tg or DR1 Tg mice have been widely used to identify and validate immunodominant T cell epitopes of synovial autoantigens, such as CII, HCgp-39, proteoglycan aggrecan, fibrinogen, and vimentin, as well as to investigate the role of posttranslational modifications on epitope binding (13, 148–151). In DR4 Tg mice immunized with citrullinated (cit)-vimentin peptide aa 59-78, specific Th1 cells and strong proliferative recall responses were detected, however, mice did not show any signs of arthritis (150). In contrast, immunization of DR4 Tg mice with human cit-fibrinogen induced vigorous citrulline-specific T and B cell responses in all DR4 Tg experimental mice and triggered arthritis, characterized by synovial hyperplasia and ankylosis, in 35% of these animals (149). Transfer of splenic lymphocytes from arthritic mice induced joint swelling in the human cit-fibrinogen–injected limb of all DR4 Tg recipient mice, but not in PBS- or unmodified human fibrinogen–injected limbs (152). The fact, that administration of cit-fibrinogen to wild-type C57BL/6 mice and immunization of DR4 Tg mice with native fibrinogen failed to induce arthritis and citrulline-specific reactivity, underscores the critical role of both SE-containing HLA-DR molecules and antigen citrullination in the development of RA (149). Another study in DR4 Tg mice corroborated that immune responses to homocitrulline-containing peptides are similarly dependent on the presence of the SE and later evolve into immune responses to citrullinated antigens (153).

With the advent of tetramer technology, it has become possible to identify, track, and characterize DR-restricted autoantigen-specific T cells in DR1 Tg and DR4 Tg mice, and to corroborate their pathogenic role in the development of RA (154–156). Activated CII-specific T cells expressing high levels of Th1, Th17 and pro-inflammatory cytokines have been detected in arthritic joints of DR1 Tg and DR4 Tg mice following immunization with CII (154, 155). Although CD4+ T cells represent only a minor population of the synovial infiltrate, they were shown to display a highly restricted TCR repertoire and limited clonality and their expansion correlated with onset and severity of arthritis as well as with anti-CII antibody levels (154, 157). It is important to note, that, at the time of first clinical signs of arthritis, activated HLA-DR4:CII(158–170) tetramer-positive cells disappeared from synovial fluid and were rarely found in blood, while they persisted in lymph nodes, suggesting that autoreactive T cells play a role particularly in the early stages of arthritis, by triggering a local immune response (154, 157). This important finding, derived from studies in DR4 Tg mice also suggests a rationale for the lack of enrichment of antigen-specific T cells in the synovial fluid of patients with established RA (171).

DR1 and DR4 Tg mice have also been used to test alternative treatments for RA, such as synthetic analog peptides, which contain substitutions in critical positions of the CII immunodominant epitope and suppress inflammatory arthritis by promoting regulatory T cell responses (172, 173). Treatment of human cit-fibrinogen-immunized DR4 Tg mice with CTLA-4Ig fusion protein, a soluble form of the CTLA-4 receptor which inhibits T cell activation by competing with CD28 for binding to the costimulatory ligand CD80/CD86 (174), restrained the activation of cit-fibrinogen-specific T cell responses, and halted the progression of arthritis (152). The fact that splenocytes from cit-fibrinogen-immunized mice treated with CTLA-4Ig were unable to transfer arthritis to recipient mice supports a direct role of activated citrulline-specific T cells in arthritis development and progression (152).

The contribution of HLA-DQ polymorphism to RA susceptibility and severity has been explored by David's group, who established mice which express transgenic DQA1^*^0301/DQB1^*^0302 (DQ8) (175). In humans, HLA-DQB1^*^03 occurs in linkage with the RA risk locus HLA-DRB1^*^04 and has been suggested to affect the clinical expression of RA (176–178). Immunization with heterologous CII induced autoreactive T and B cell response and severe inflammatory arthritis in 70% of DQ8 Tg animals (179), while of mice expressing a protective DQ6 Tg, only 14% presented mild arthritis and 60% of Tg mice with mixed DQ8/DQ6 haplotype developed moderate CIA in response to CII (180). DQ8 Tg mice with deleted CD4 did not develop arthritis, while CD8 deficient DQ8 Tg mice developed severe CIA along with increased autoantibody levels, suggesting that CD4+ T cells, but not CD8+ T cells are indispensable for initiation of CIA (181). In the absence of B cells, DQ8 mice failed to develop CIA, suggesting that B cells are not only important for autoantibody production, but also for antigen presentation to autoreactive T cells (146). To simulate the situation in humans, where HLA-DR and -DQ occur in linkage, Taneja and coworkers established mice that express DR4 (either RA-susceptible ^*^0401 or RA-resistant ^*^0402) along with DQ8 transgene (182). Indeed, CIA incidence was increased in ^*^0401.DQ8 double Tg mice compared to single Tg mice, while ^*^0402.DQ8 Tg mice were resistant to CIA (182).

The link between smoking and the emergence of RA in individuals with an RA-associated HLA haplotype has been reproduced in DR4 and DQ8 Tg mice (183). Exposure to cigarette smoke augmented PAD enzyme expression and enhanced pro-inflammatory Th1 and antibody responses to native and citrullinated CII and vimentin in both DR4 and DQ8 Tg mice, while promoting Th2/Treg responses in Tg mice that expressed protective DRB1^*^0402 (183, 184). An association between periodontal disease and RA has been studied on DR1 Tg mice, in which oral exposure to *Porphyromonas gingivalis* led to increased percentage of Th17 cells in blood and lymph nodes, systemic pro-inflammatory cytokine response, loss of bone density, and generation of anti-citrullinated protein antibodies (185).

### Human Autoantigen Transgenic Mouse Models

The breakdown of immune tolerance to endogenous cartilage antigens is a central element in the development of RA. Immunization of CIA-susceptible mouse strains with non-self (e.g., human, bovine, chicken, or rat) CII induces severe arthritis whereas mice are less prone to CIA induction with self (mouse) CII (186). This is due to minor but decisive structural differences between mouse and human CII, which are located in the immunodominant T cell epitope CII(158–170, 187, 188), containing glutamic acid at position 266 in human (as well as bovine, chicken and rat) CII, whereas in mouse CII there is an aspartic acid instead (189).

In mice which express the entire human CII protein, or its immunodominant human (h)CII(158–170, 187, 188) epitope within a mutated mouse CII (MMC) protein, only autoreactive T cells should become activated upon immunization with human or rat CII. Indeed, although hCII Tg mice with cartilage-restricted expression of hCII generally mounted autoreactive T and B cell responses following immunization with hCII, only 10% of these mice developed arthritis (186). In this model, T cell tolerance was shown to depend on the expression level of hCII and its immunodominant epitope (186). In B10 mice expressing DR4 transgene together with either hCII or MMC, murine DR4-restricted T cells were tolerized against self-CII, and thus these mice were uniformly resistant to CIA (151, 190). In contrast, T cell tolerance to self-CII was incomplete in MMC.DR4 Tg mice with C3H background, rendering these mice prone to develop CIA (151). Tolerance to self-CII could be broken in B10.DR4 mice expressing MMC, but not hCII Tg, by introducing a mutation in the *Ncf1* gene, which encodes a subunit of the NADPH oxidase complex, resulting in reduced ROS production (151, 191).

### Human Autoantigen-Specific T Cell Receptor Transgenic Mouse Models

Mice that express a transgenic TCRαβ specific for an arthritogenic epitope of human autoantigen, such as CII or proteoglycan aggrecan, were used to gain more insight into the role of antigen-specific T cells in the development of autoimmune arthritis (192, 193). In such TCR Tg mice, more than 90% of CD4+ T cells express the antigen epitope-specific TCR transgene (192, 193).

TCR Tg mice, carrying the rearranged V_α_11.1 and V_β_8.3 chain encoding genes specific to bovine CII, respond vigorously to stimulation with bovine CII or its immunodominant determinant CII(158–164, 188) *in vitro*, but do not develop spontaneous arthritis (192, 194). However, an accelerated onset and increased severity of arthritis was observed in CII-specific TCR Tg mice after immunization with CII in CFA, as compared to their non-Tg littermates (192, 194). Interestingly, Treg cell clones generated from splenocytes of CII-specific TCR Tg mice reduced the proliferation of CII-specific effector T cells *in vivo* and decreased the incidence and clinical symptoms of arthritis after adoptive transfer in CIA and collagen antibody-induced arthritis models (195).

Another TCR-Tg mouse model was generated by introducing TCR V_α_1.1 and V_β_4 chains of a T cell hybridoma with MHC class II-restricted specificity for the immunodominant epitope (5/4E8) of human cartilage proteoglycan aggrecan into PGIA-susceptible BALB/c strain (193). In these TCR Tg mice, a single dose of human proteoglycan, even in the absence of adjuvant, produced disease (193), which is in contrast to the conventional PGIA model, that requires multiple immunizations with human proteoglycan in adjuvant (12).

An elegant strategy has been to combine the expression of RA-associated DR1 and a transgenic V_α_2/V_β_8.1 TCR, recognizing the immunodominant determinant of bovine and human CII (196). These double Tg mice developed an accelerated and more severe form of CIA than their DR1 Tg littermates and provide a more reliable tool for testing novel therapeutic approaches (196).

TCR-Tg models support the hypothesis that antigen-specific T cells play a critical role in the initiation of arthritis. However, important limitations are the restriction to a single autoantigen epitope and the decrease of the CD8+ T cell pool in these mice (192–194).

In an ideal Tg mouse model of autoimmune arthritis, combined expression of RA-susceptible HLA-DR molecules, an RA-relevant autoantigen and a specific TCR, recognizing the immunodominant epitope of this autoantigen, would lead to spontaneous breakdown of self-tolerance and the development of arthritis. This has not been achieved so far, however, studies in Tg mouse models of RA have underscored the critical role of the SE, post-translational modification of antigen and self-reactive T cells in the development and progression of RA and contributed substantially to the identification and validation of immunodominant autoantigens and its T cell epitopes. Thus, Tg mice represent a decisive step forward in RA research and have undoubtedly enhanced the predictive value of mouse models in preclinical tests. However, it must be considered, that many of the herein presented models have been only established in one laboratory or published once. The poor reproducibility under different laboratory conditions might be at least partially explained by distinct microbial communities. Another problem is that human DR transgenes have been shown to be poorly expressed by DCs in some strains, resulting in poor antigen presentation. Finally, Tg mouse models still bear the same limitation as conventional models of RA-like disease, that is, inflammation is driven by the murine immune system that ectopically expresses SE-containing HLA class II molecules or a specific TCR but might contribute in a different way to the development of the disease.

## Mouse/Human Chimeras as Humanized Models of Rheumatoid Arthritis

### Immunodeficient Mice as a Platform for the Engraftment of Human Cells and Tissues

An important advance in the generation of a preclinical mouse model of RA would be the establishment of a functional human immune system or some of its components in mice, in which they mount autoimmune responses and clinical features of RA. For this purpose, human hematopoietic stem and progenitor cells (HSC), peripheral blood mononuclear cells (PBMC), or tissue have been engrafted into immunodeficient mice, which lack the ability to reject xenografts and thus enable stable reconstitution with human cells (Figure 1).

Important achievements of the last decades have paved the way for the stable engraftment of human cells within the murine host. The *Prkdc*^*scid*^ (protein kinase, DNA activated, catalytic polypeptide) mutation in CB17 mice, commonly denoted “SCID” (severe combined immunodeficiency) (197), results in reduced numbers of functional T and B cells and thus enables limited and transient engraftment of human PBMCs, HSCs and fetal hematopoietic tissues (198–200). However, spontaneous generation of mouse T and B cells with aging, referred to as leakiness, as well as high levels of host natural killer (NK) cells and innate immune activity impede the stable engraftment of human cells and tissues in SCID mice (29). Alternatively, targeted mutations of the recombination-activating genes 1 (*Rag1*) and *Rag2* prevent the development of functional T and B cells in mice without causing leakiness (201, 202). Nevertheless, Rag1/2-deficient mice retain high levels of NK-cell activity, allowing only limited engraftment of human HSC (29).

An important step forward was accomplished by crossing NOD and SCID strains (203). NOD-SCID mice display additional defects in innate immunity, including the absence of complement C5, impaired macrophage cytokine production, antigen presentation and NK cell function (29, 203, 204). Although they have increased engraftment of human HSCs and PBMCs (205, 206), the limitations of NOD-SCID mice include relatively short life span due to thymic lymphomas, and residual activity of NK cells and innate immunity (29).

A decisive breakthrough was achieved by the generation of immunodeficient mice with a mutation in the *Il2rg* gene (207–210), encoding IL-2 receptor γ subunit (IL-2Rγ), also denominated gamma-chain, required for IL-2, IL-4, IL-7, IL-9, IL-15, and IL-21 signaling (211). An absent or truncated IL-2Rγ-chain leads to defective T and B cell development, affects innate immunity and completely abolishes NK cell generation (212, 213). The main immunodeficient mouse strains bearing the *IL2rg* mutation are NOD-SCID *Il2rg*^*null*^, including NOD-SCID *Il2rg*^*tm*1*Wjl*^ (NSG) and NOD-SCID *Il2rg*^*tm*1*Sug*^ (NOG), as well as NOD-*Rag1*^*null*^ *Il2rg*^*null*^ (NRG) and BALB/c-*Rag2*^*null*^ *Il2rg*^*null*^ (BRG) (208, 209, 214, 215). These strains support high engraftment of human tissue, HSCs and PBMCs (207, 208, 216), without the need for previous myeloablation through irradiation or drugs, thus reducing the required donor cell number and prolonging survival of humanized mice (217). Engrafted HSCs differentiate into multiple lineages of human cells, including erythrocytes, platelets, T and B lymphocytes, NK cells, DCs, monocytes/macrophages and granulocytes (207–209, 215, 218). Reconstitution of the human immune system is most efficient, when CD34+ HSCs from cord blood or fetal liver (rather than from adult peripheral blood) are injected into newborn mice, where both donor cells and recipient are set for development and expansion of the hematopoietic system (215, 219). Co-transplantation of HSCs with fetal liver and/or fetal thymus (abbreviated BLT for bone marrow, liver, thymus) further improves the systemic repopulation with multilineage human cells, by providing an autologous thymic environment for proper T cell development (220, 221). The functionality of the reestablished human immune system in immunodeficient mice has been demonstrated by the presence of lymphoid tissues and the capacity to mount adaptive immune responses, including T cell-dependent antibody responses, cell-mediated cytotoxicity and delayed type hypersensitivity reactions (207, 217–219, 222).

However, absence of the IL-2Rγ-chain results in a lack of some cytokines which are cross-reactive between human and mouse and are required for human cell differentiation and survival within the host (29, 223). Furthermore, cross-reactivity between murine and human cytokines does not necessarily imply a biological function, as demonstrated by the example of B lymphocyte survival factor/stimulator BAFF (224). To overcome this issue, human cytokines and growth factors, including IL-7, IL-6, BAFF, thrombopoietin, FLT3-ligand, IL-12, granulocyte-macrophage colony-stimulating factor and IL-3 can be either administered or provided by transgenic expression (208, 224–229).

A major obstacle to using humanized mice as a preclinical model is their susceptibility to xenogeneic graft-vs.-host disease (GvHD), which impedes the development of chronic disease (230). This applies rather to mice that have been engrafted with BLT or human PBMCs than to HSC-engrafted mice. In the first two models, mature CD4+ T cells have been educated in human thymic stroma and are therefore not tolerized to the murine antigenic environment, which leads to rapid-onset xenogeneic GvHD (231). Engraftment of human cells and GvHD onset and severity vary between donors and seem to depend on the dose of CD4+ and CD8+ T cells as well as of naïve CD4+ T cells within the transferred human cells (232, 233). In contrast, higher percentages of Treg cells delay xenogeneic GvHD (234). Importantly, human T cells in HSC-reconstituted mice are selected on murine MHC class II (H2) molecules in the mouse thymus and therefore might not be able to recognize antigens presented in the context of HLA-DR by human antigen-presenting cells in the periphery. This affects the induction of efficient immune responses, resulting in reduced Th1 activity and insufficient interactions between human T and B cells which are required for class-switch recombination (235, 236).

A significant improvement has been accomplished by the introduction of human HLA class II molecules into immunodeficient mouse strains. Transgenic expression of HLA-DR4 in NRG mice enabled proper development of CD4+ T cells and completely functional B cells from infused HSCs of HLA-DR-matched donors (237). The additional removal of murine MHC class II molecules, which are the main target of human CD4+ T cell-mediated GvHD responses further improved the generation of human antigen-specific immune responses in immunodeficient mice reconstituted with human cells (238, 239), while reducing the risk of xenogeneic GvHD (240).

### Attempts to Establish Autoimmune Diseases in Chimeric Humanized Mouse Models

While humanized mouse models based on mouse/human chimeras have been widely used for studies on cancer, human-specific infectious diseases and transplantation (216, 236, 241, 242), their great potential to recapitulate human autoimmune disorders has only recently been recognized and explored (243).

It has been shown that transfer of human HSCs with a psoriasis-prone HLA haplotype provokes a related disease in NSG mice (244). Recently it has been demonstrated that reconstitution of NSG-Ab^0^ DR1 mice (lacking murine MHC class II (Ab^0^) and expressing transgenic HLA-DR1) with HSCs from a patient with immunodysregulation polyendocrinopathy enteropathy X-linked (IPEX) syndrome, associated with FOXP3 dysfunction, spontaneously develop lethal autoimmune disease, involving multiple organs and the production of autoantibodies (245). In another study, autoimmune disease characterized by hepatitis, weight loss and anti-nuclear antibodies was induced in HSC-reconstituted NSG mice by inhibition of CTLA-4, a critical molecule for Treg suppressive function (246). A spontaneous humanized model of type 1 diabetes (T1D)-like disease was established by engrafting HSCs into NRG-Akita mice, which develop hyperglycemia due to a mutation in the insulin 2 gene (214). In NSG.DQ8 Tg mice reconstituted with human DQ8+ HSCs and fetal thymus, transfer of autologous human CD4+ T cells expressing an DQ8/insulin B chain peptide-specific TCR and immunization with the corresponding peptide induced hyperglycemia and diabetes (247).

Other investigators engrafted patient-derived PBMCs into immunodeficient mice to reconstitute T cell-mediated autoimmune disorders, such as Sjögren's Syndrome, Systemic Lupus Erythematosus (SLE), or T1D, involving the production of autoantibodies and tissue-specific autoreactivity (243, 248, 249). Engraftment of BRG mice with PBMC from SLE patients caused autoimmune-like disease with donor-dependent severity, characterized by nephritis, proteinuria, deposits of human IgG in kidneys and shorter life span compared to mice engrafted with PBMCs from healthy subjects (249). Similarly, engraftment of NSG.A2 mice, expressing T1D-associated HLA-A^*^0201 (A2) molecules, with HLA-A2 matched PBMCs from T1D patients resulted in islet infiltration by specific CD8+ T cells (243). Insulitis and pancreatic β cell death, the characteristic hallmarks of T1D, were also induced by the transfer of autoreactive CD4+ T cell lines from T1D donors into NSG.DR4 mice (250). In these models, spontaneous development and severity of autoimmune-like disease are donor-dependent and mice that have been reconstituted with PBMCs from healthy subjects fail to display an autoimmune phenotype (243, 249).

**Figure 3:**
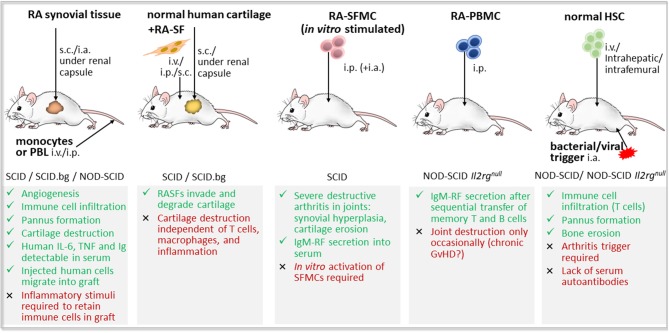
Humanized mouse models of rheumatoid arthritis based on the engraftment of human cells and/or tissues in immunodeficient mice. RA-like features (green color) and drawbacks (red color) of the five main approaches for the development of a humanized mouse model of rheumatoid arthritis (RA). First, transplantation of RA synovial tissue and eventual co-injection of peripheral blood lymphocytes (PBL) or monocyte cell line; Second, co-implantation of normal human cartilage and RA patient-derived synovial fibroblasts (RA-SF); Third, injection of synovial fluid mononuclear cells (SFMCs) from RA patients, either stimulated *in vitro* or not; Fourth, inoculation of RA patient-derived peripheral blood mononuclear cells (PBMCs); and fifth, induction of RA-like disease by viral or bacterial triggers in immunodeficient mice that have been reconstituted with human hematopoietic stem and progenitor cells (HSCs). GvHD, graft vs. host disease; i.a., intraarticular; *Il2rg*^*null*^, defective interleukin 2 receptor γ chain; i.p., intraperitoneal; i.v., intravenously; NOD, non-obese diabetic mouse; s.c., subcutaneously; SCID, severe combined immunodeficiency; SCID.bg, SCID mouse with reduced NK cell activity due to *beige* mutation.

Five different approaches have been pursued to generate humanized mouse models of RA, using immunodeficient mice as host for (i) RA synovial tissue (251, 252), (ii) RA patient-derived synovial fibroblasts co-transplanted with normal human cartilage (253, 254), (iii) synovial fluid mononuclear cells (SFMCs) (255, 256), (iv) RA patient-derived PBMCs (233, 256), or (v) HSCs (257, 258) (Figure 3).

### Transplantation of Rheumatoid Arthritis Synovial Tissue Into Immunodeficient Mice

It has been demonstrated that implanted human rheumatoid synovium maintains its histological and phenotypic properties in SCID mice and survives for more than 6 months (251). Synovial tissue transplants become vascularized by human endothelial cells and human vessels were connected to mouse vasculature (259, 260). To generate RA synovium/SCID mouse chimeras, pieces of rheumatoid synovium were placed into SCID mice subcutaneously (260, 261), under the renal capsule (251, 262) or into joints (263, 264). As a result, RA-like disease, characterized by inflammation, angiogenesis, pannus formation and cartilage infiltration by fibroblast-like cells developed (260, 262, 263). The implanted rheumatoid synovium even invaded and destroyed co-implanted normal human cartilage (262). Importantly, only synovial tissue from RA patients, but not healthy synovium, induced pannus formation and destruction of bone and cartilage, when transplanted into joints of beige SCID mice (263). Both human and murine macrophages as well as murine granulocytes contribute to synovial inflammation in this model (263, 264). Human TNF, IL-6 and all Ig subclasses were detected in the serum of RA synovium-grafted animals (260, 265, 266). However, in the absence of inflammatory stimuli present in the RA joint, human synovium returns to a “resting state” after transplantation into immunodeficient mice, as demonstrated by the decreased expression of cell adhesion molecules ICAM-1 and VCAM-1 and emigration of mononuclear cells (259, 267). To overcome the issue of decreased lymphocyte numbers within RA synovial grafts, human allogeneic or autologous PBMCs or T cells were injected into the peritoneal cavity, tail vein, or directly into the graft of RA synovium SCID mouse chimeras (251, 268, 269). However, only 1–5% of intravenously injected peripheral blood lymphocytes (PBL) were shown to reach the graft, while most human PBL were sequestered in spleen and liver (259). Other studies demonstrated that injected T cells specifically migrate into synovial grafts, though migration was not specific to the synovial origin or inflammatory state of the human graft (251, 260). Co-administration of pro-inflammatory mediators, such as TNF, the acute-phase protein serum amyloid A and IL-15, have been shown to play a critical role in maintaining an inflammatory milieu, which retains mononuclear cells within and enhances lymphocyte and monocyte migration toward human synovial grafts (252, 267–269). Elegant studies by Weyand's group, in which either T cells or B cells were depleted from the RA synovial grafts in NOD-SCID mice, revealed that Th1 cells drive pro-inflammatory cytokine and tissue-degrading enzyme expression by synovial macrophages, and underlined a critical role of B cells in T cell activation and synovial inflammation (270, 271).

The RA synovium/SCID mouse chimera model has been widely used to study the properties of RA synovium *in vivo*, and to explore the effect of inflammatory mediators and its inhibitors on angiogenesis, cytokine secretion, and inflammatory cell infiltration (252, 266, 268, 269). The RA synovium/SCID mouse model was also used to investigate the mechanisms of action of anti-rheumatic drugs *in vivo* and to test novel biologic agents for the treatment of RA, such as monoclonal antibodies directed against human IL-6R, CD147 or Fas, as well as T and B cell–related therapies using CTLA-4Ig, anti-CD20, and anti–IL-17 antibodies (266, 272–275). For example, methotrexate decreased the number of inflammatory cells in RA synovial grafts through apoptosis (272), and anti-TNF antibodies, although reducing synovial inflammation, did not prevent bone and articular cartilage damage (276). This model also demonstrated inefficacy of anti-IL-1 and CTLA-4Ig therapy in the reduction of cellular infiltration and IL-6 secretion, and showed selective decreases in IL-6 secretion by anti-IL-17 only in those RA synovial tissues which contain high numbers of T cells (266).

Co-implantation of normal human cartilage with RA synovium into SCID mice resulted in pannus-like formation, cartilage invasion and perichondrocytic degradation for an extended period (261, 262). Intra-graft injection of IL-10 inhibited cartilage degradation and decreased ICAM-1 expression in and PBMC traffic toward RA synovial tissue in this model (261). In another approach, tissue derived from human RA pannus was implanted subcutaneously together with a slice of dentin into SCID mice (265). This work elucidated that only concomitant treatment with methotrexate and the TNF inhibitor infliximab suppressed pit formation in the dentin slice and thus detains bone destruction (265).

### Implantation of RA Synovial Fibroblasts and Human Cartilage Into Immunodeficient Mice

In the SCID mouse co-implantation model of RA, synovial fibroblasts from RA patients (RASF) and normal cartilage were placed together in a gel sponge either subcutaneously or under the renal capsule (253, 254). The gel sponge replaced the synovial matrix as carrier for synovial fibroblasts, providing an environment devoid of stimulatory or inhibitory effects of other cellular and matrix components (253). This allowed study of the invasion of human RASF into human cartilage in a non-inflammatory environment. Using this model, RASFs were shown to invade and destroy human articular cartilage, independent of T cells, macrophages, and inflammation (253, 277). Activated RASFs specifically migrated toward, invaded and degraded implanted human cartilage, mirroring the progression from oligo- to polyarticular disease (254). However, due to the absence of inflammation and (antigen-specific) T and B cell responses, this SCID mouse co-implantation model of RA appears to rather reflect a facet of the disease, than the complete process of RA development.

### Transfer of Rheumatoid Arthritis Peripheral Blood- or Synovium-Derived Mononuclear Cells Into Immunodeficient Mice

A first attempt to engraft mononuclear cells from peripheral blood, synovial fluid or synovial tissue of RA patients into immunodeficient mice was made in 1990 by Tighe and colleagues (255). Transferred PBMCs and SFMCs continued producing human IgG and IgM in SCID host mice, and IgM rheumatoid factor could be detected in mouse serum for more than 20 weeks after human cell engraftment (255). Later, Sakata and coworkers showed that previously stimulated synovial fluid-derived T cells from RA patients, simultaneously injected into knee joint and peritoneal cavity, caused severe destructive arthritis in SCID mice (256). Interestingly, arthritis occurred not only in the joint that received the cell injection but also in other joints, suggesting RA-like polyarthritis (256). In contrast, transfer of unstimulated synovial fluid-derived T cells or *in vitro*-activated PBMCs from RA patients failed to trigger arthritis in SCID mice (256, 263).

Almost two decades later, Ishikawa and colleagues engrafted RA patient-derived PBMCs into NOG immunodeficient mice (233). Since transfer of PBMC often causes lethal GvHD, the naïve CD4+ T cell fraction was removed from PBMCs, and RA patient-derived CD4+ memory T cells and B cells were transferred sequentially, resulting in sustained production of IgM rheumatoid factor autoantibodies in human cell-engrafted NOG mice (233). However, only some mice reconstituted with RA patient-derived PBMCs displayed histological joint destruction, which was difficult to distinguish from alterations due to chronic GvHD (233). Nonetheless, it has been proven that human T cells engrafted in NOD-*scid IL2rg*^*null*^ mice migrate to air pouches containing RA synovial fluid and this recruitment was abolished by CXCR3 agonist (278).

The development of RA-like disease in immunodeficient mice engrafted with human cells seems to critically depend on the origin (peripheral blood vs. synovial fluid) and previous *ex vivo*-activation of transferred cells through mitogen or autoantigen, as well as on cell number and route of administration (intra-articular, intraperitoneal, or intravenous). Unlike other models of autoimmune diseases (243, 248, 249), injection of PBMCs of RA patients, even when activated, was insufficient to induce arthritis (233, 256, 263), indicating that pathogenic T cells might be concentrated in synovial fluid and tissue. Previous *ex vivo* activation of synovial fluid-derived T cells appears to facilitate the establishment of arthritis in immunodeficient mice through expansion of antigen-specific arthritogenic T cell clones and concomitant induction of growth factors such as IL-2 (256). Sole intra-articular injection of small numbers of mitogen-stimulated SFMCs or CII-specific T cell lines was not sufficient to induce joint destruction in beige SCID mice (263), while combination of systemic and local administration of synovial fluid-derived cells appeared to concert inflammatory cell infiltration and local synovial cell proliferation and thus promote the development of RA-like lesions (256). It is of note that at 4 weeks after engraftment into NOD-*scid IL2rg*^*null*^ mice, almost all human cells were activated T cells and thus, it must be considered that the absence of myeloid cells may affect the development of a RA-like disease (278). The supply of antigen-presenting cells e.g., through repetitive injections of (auto-)antigen-loaded DCs or B cells, might be an option to circumvent this issue (158, 187, 188, 233).

### Hematopoietic Stem Cell-Engrafted Mouse Models of Rheumatoid Arthritis

With the advent of immunodeficient mouse models that enable the complete reconstitution of the human immune system through engraftment of HSCs, researchers have also exploited this option to establish humanized mouse models of RA.

Injection of *Chlamydia trachomatis* into knee joints of NOD-SCID mice, that had been previously irradiated, treated with anti-CD122 Ab to block the IL-2R and repopulated with CD34+ bone marrow-derived HSCs from osteoarthritis patients, induced synovial inflammation with a predominance of human CD68+ macrophages (159). The main limitation of this model is the absence of mature human CD4+ and CD8+ T lymphocytes (159). To overcome this issue, later models used umbilical cord blood-derived HSCs which were able to give rise to all immune cell types, instead of HSCs from adult peripheral blood with restricted hematopoietic potential (210, 219).

Kuwana and coworkers used NOG mice transplanted with cord blood-derived HSCs to investigate the contribution of Epstein-Barr virus (EBV) infection to the development of RA (257). Erosive arthritis of the major joints, histologically characterized by pannus formation, bone marrow edema, synovial membrane proliferation and infiltration of inflammatory cells, mainly T cells, occurred in 65% of low dose EBV-infected, but not in non-infected, humanized NOG mice (257). However, rheumatoid factor and ACPA autoantibodies were not detected in peripheral blood of these mice (257). In another approach, acute inflammatory arthritis was induced in HSC-reconstituted NSG mice, through intra-articular injection of CFA (258). These mice developed clinical and histological signs of arthritis, such as swelling, erythema, decrease of function, immune cell infiltration, and bone erosion (258). Elimination of murine neutrophils by pre-treatment with anti-Gr-1 antibody did not affect arthritis development and human leukocytes were detected in inflammatory infiltrates, indicating that RA-like disease was mediated by engrafted human cells infiltrating the joint (258). Treatment with the TNF inhibitor Etanercept prior to the induction of CFA-triggered arthritis was shown to decrease scores of pannus formation, inflammation and bone erosion, as well as human cell counts in joints (258).

So far only healthy donor-derived HSCs have been used to establish the human immune system in immunodeficient mice. An ideal model of RA pathogenesis would utilize HSCs derived from RA patients, which has been non-feasible. On the other hand, stem cells obtained from adult peripheral blood have proved to be unsuitable to completely reconstitute the immune system in immunodeficient mice (159, 219, 258). Induced pluripotent HSCs or cord blood-derived HSCs from relatives or offspring of RA patients, which might be equally predisposed to develop RA, or healthy individuals with RA-susceptible HLA-DR haplotype, might serve as source of HSCs for engraftment, but are difficult to obtain. Moreover, RA models based on immunodeficient mice, whose immune system has been reconstituted through engraftment of human stem cells, require a local inflammatory stimulus in the joint, such as viral or bacterial infection, to induce immigration of inflammatory cells and trigger RA-like disease.

Taken together, the ultimate humanized model of RA, mirroring all aspects of this chronic inflammatory autoimmune disease does not yet exist. Since T cells play a minor role in synovial tissue implant and RASF transfer models, these models are less suitable for testing cell-based therapeutic strategies targeting autoreactive T cells. Autoreactive lymphocytes are found in peripheral blood and, to a much higher extent, in synovial fluid and tissue of RA patients, which might therefore be the cellular source of choice to recapitulate arthritis in immunodeficient mice. However, *in vitro* T cell activation prior to transfer seems to be necessary, raising the question of antigen-specificity (256). Finally, development of arthritis-like symptoms in immunodeficient mice that have been engrafted with healthy donor HSCs requires pathogenic stimuli and thus does not involve RA-associated autoantigens (159, 258), unless HSC donors expressing RA risk alleles are used for the reconstitution of the human immune system.

## The Challenge of Translating Cell-Based Therapies for Rheumatoid Arthritis in Humanized Mice

**Figure 4:**
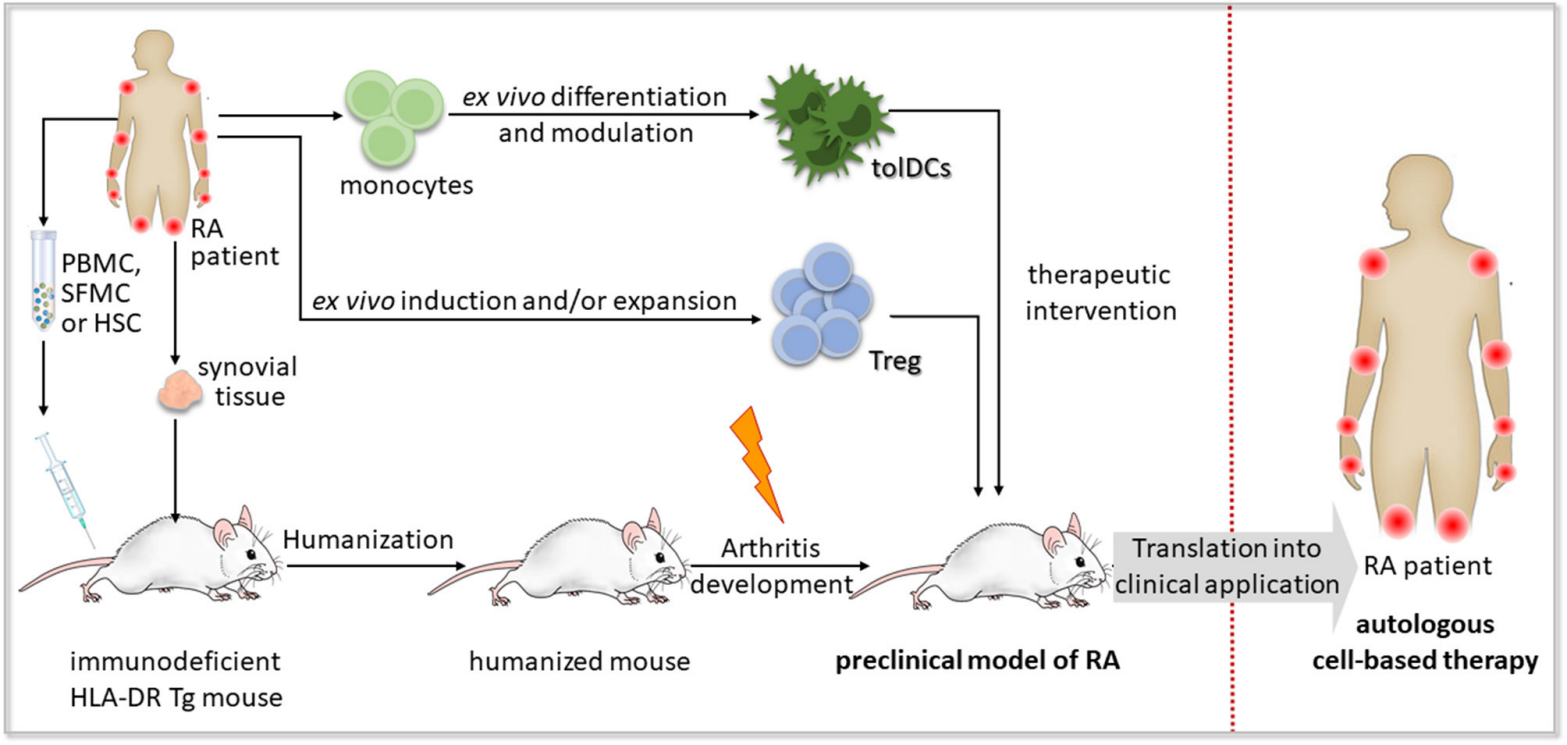
Concept of preclinical testing of cell-based immunotherapy for rheumatoid arthritis in humanized mice. A humanized mouse model of rheumatoid arthritis (RA) could be established by the engraftment of human synovial tissue, hematopoietic stem and progenitor cells (HSCs), peripheral blood mononuclear cells (PBMCs) or synovial fluid mononuclear cells (SFMCs) from an RA patient bearing HLA-DR risk alleles such as HLA-DR*0401 or HLA-DR*0101 into immunodeficient mice expressing the respective transgenic (Tg) HLA-DR molecules. Autoimmune-like disease, if not developed spontaneously, might be induced by an additional trigger, such as RA-associated autoantigens or autoreactive T cell clones. The obtained humanized mouse model of RA enables preclinical testing of cell-based immunotherapeutic approaches applying autologous regulatory T (Treg) cells or tolerogenic dendritic cells (tolDCs) to recover self-tolerance, before their transfer to clinical application in RA patients.

Humanized mouse models, particularly those reconstituted with human leukocyte populations, provide a promising tool for preclinical testing of novel therapeutic approaches, such as cell-based immunotherapy (Figure 4).

The clinical response of individual patients toward autologous cell-based immunotherapy has already been tested in humanized mouse models of diverse human cancers (160, 241). Adoptively transferred Treg cells, converted from CD4+ T cells through enforced expression of IL-10, were demonstrated to exert anti-tumor and anti-leukemic effects in NSG mice reconstituted with ALL-CM or THP-1 leukemia cell lines, through granzyme B-mediated lysis of myeloid tumor cells in a HLA class I-dependent but antigen-independent manner (161). These CD4^IL10^ cells were also able to contribute to graft vs. leukemia activity of injected allogeneic PBMCs, while preventing xenogeneic GvHD (161). Administration of autologous mature DCs, pulsed with tumor-associated antigen MART1 or WT1, was shown to induce antigen-specific cytotoxic T cell responses in NSG mice reconstituted with human PBL (158, 187). In a humanized mouse model of breast cancer, established by injecting breast cancer stem cells into the mammary fat pad of HSC-reconstituted NOD-SCID mice, the application of autologous DCs loaded with breast cancer stem cell antigen reduced tumor size and prolonged survival (162).

Adoptive immunotherapy with human DCs, engineered to be long-lived and to co-express high levels of human IFN-α, human GM-CSF and cytomegalovirus pp65 antigen, regenerated immunity *de novo* in NRG mice after human allogeneic HSC transplantation (163, 164). *In vivo* targeting of Epstein-Barr virus nuclear antigen 1 (EBNA1) to the endocytic receptor DEC-205 expressed by CD141+ DCs, stimulated antigen-specific CD4+ T cell responses in NSG mice reconstituted with human fetal liver-derived CD34+ HSCs (165).

Promising cell-based approaches have also been achieved in humanized mouse models of transplant rejection. Injection of freshly isolated or *ex vivo*-expanded human CD4+ or CD8+ Treg cells into immunodeficient mice, that had been transplanted with human skin and reconstituted with allogeneic PBMCs, supported long-term survival of skin allografts (166–168). Similarly, transfer of *ex vivo* expanded autologous Treg cells prevented rejection of islet xenografts in mice reconstituted with human PBMCs or CD34+ HSCs by inhibiting immune cell infiltration and T effector cell differentiation in an IL-10 dependent manner (169, 170, 279).

In a humanized model of allergic airway disease, consisting of NSG mice reconstituted with PBMC from patients with allergic asthma and sensitized to birch pollen in the presence of IL-4, administration of autologous Treg cells or *in vivo* Treg induction through sGARP or polyclonal activation via gp120, abrogated airway hyperresponsiveness and reduced airway inflammation in the lung in a TGF-β receptor 2-dependent manner (280, 281). In a similar model of allergen-specific gut inflammation, induced by rectal challenge with birch, grass pollen, or dust mite allergen, adoptive transfer of activated Treg cells decreased allergen-specific Th2 responses and IgE secretion (282).

Despite the great potential of humanized mice as preclinical models (241, 283, 284), there are only few studies that use mouse/human chimera to explore the efficacy of cell-based immunotherapies for autoimmune diseases *in vivo*, none of them in RA.

In humanized models of autoimmune T1D, autoantigen-specific vaccination strategies have been described, which aim at inducing Treg cells that prevent the destruction of insulin-producing β cells (285, 286). Systemic delivery of nanoparticles, coated with autoimmune disease-relevant peptides bound to MHC class II molecules, promoted the *in vivo* generation and expansion of antigen-specific FOXP3-CD49b+LAG-3+ Treg cells in different humanized mouse models, including NSG mice reconstituted with T1D patient-derived CD8+ T cell-depleted PBMCs, and ameliorated clinical and pathological signs of CIA and experimental autoimmune encephalomyelitis in *HLA-DR4-IE* Tg mice (285). In another study, sub-immunogenic vaccination of human HSC-engrafted NSG.DQ8 Tg mice with agonistic mimetopes of the T1D-relevant insulin B-chain epitope induced insulin-specific FOXP3+ Treg cells *in vivo* (286).

To explore therapeutic strategies to overcome the resistance of effector T cells toward Treg-mediated suppression in multiple sclerosis (MS) patients, a humanized model based on newborn Rag2^−/−^γ^−/−^ mice engrafted with CD25-depleted PBMCs from different MS patients was applied (287). Transfer of gp120-activated Treg cells from healthy subjects into these mice prevented GvHD only when engrafted PBMCs were derived from MS patients that received disease-modifying therapy but not from those being therapy-naïve (287).

In a humanized model of myasthenia gravis (MG), generated by subcutaneous engraftment of thymic MG fragments into NSG mice, administration of *in vitro*-preconditioned human mesenchymal stem cells improved the disease by decreasing the serum level of acetylcholine receptor (AChR)-specific autoantibodies and restoring AChR expression at the muscle endplate (288).

Novel therapeutics for RA have been tested so far only in DR1 and DR4 Tg mice. Particularly, the capacity of CII analog peptide has been demonstrated to induce *in vivo* antigen-specific inhibitory T cells capable of suppressing CIA dependent on IL-10 and IL-4 secretion (173, 289). Nevertheless, it seems to be only a matter of time until humanized chimeric mouse models of RA will be available for preclinical testing of cell-based therapeutic strategies. But there are still some important obstacles to overcome (Table 2).

**Table 2:** Challenges of recapitulating rheumatoid arthritis in chimeric humanized mice.

**Problems**	**Possible solutions**
Recipient gender-dependent engraftment of human cell populations	•Use of female mice only
Donor-dependent variations in PBMC engraftment	•Transfer of defined cell populations rather than whole PBMCs •Personalized model
Xenogeneic GvHD in PBMC-engrafted mice prevents development of a chronic disease model	•Use of human HSCs as cellular graft •Removal of naïve CD4+ T cells from human cell graft •Deletion of murine MHC class II •Expression of transgenic HLA-DR1 or -DR4 and engraftment of HLA-DR-matched donor cells
Disappearance of myeloid APCs in PBMC-engrafted mice	•Sequential transfer of (antigen-pulsed) APCs •Supply of survival factors, such as human GM-CSF
Difficulties in establishing autoimmune disease	•Removal of Treg cells from human cell graft •Transfer of autoreactive CD4+ T cell clones •Transfer of SFMCs and/or synovial tissue from RA patients
Poor autoantibody production	•Sequential transfer of B cells •Administration of BAFF
Unknown trigger of autoimmunity	•Transplantation of synovial tissue or administration of synovial fluid of RA patients

*APCs, antigen-presenting cells; BAFF, B cell activating factor; GM-CSF, granulocyte macrophage colony-stimulating factor; GvHD, graft vs. host disease; HLA-DR, human leukocyte antigen DR; HSCs, hematopoietic stem and progenitor cells; MHC, major histocompatibility complex; PBMCs, peripheral blood mononuclear cells; SFMC, synovial fluid mononuclear cells; Treg, regulatory T cell*.

Many of the RA models have been difficult to reproduce by other laboratories, requiring profound characterization of environmental und genetic susceptibility factors that interfere with, or promote, RA-like disease in humanized mice.

One major problem in humanized mice is that long-term evaluation of transferred human cells and the establishment of chronic disease is hampered by the development of xenogeneic GvHD, induced by cross-species interactions between mouse macrophages and human CD4+ T cells in skin and lymphatic tissues (290). HSC-reconstituted mice are less susceptible to GvHD than PBMC-engrafted mice, since T cell selection in this model occurs in the murine thymus. However, PBMCs might be the better choice for the establishment of an RA model, as autoimmunity develops due to the loss of peripheral but not central tolerance. Since it has been postulated that GvHD in human PBMC-engrafted SCID mice results from anti-mouse MHC class II reactivity of human CD4+ T cells (291), a possible solution would be to use immunodeficient mice that lack murine MHC class II molecules as platform for the engraftment of human cells (238). Additional expression of transgenic human RA-associated DR4 or DR1 molecules might further improve long-term engraftment of cells and tissues from HLA-DR-matched human donors (237, 239, 245). Alternatively, removal of the naïve CD4+ T cell fraction from transferred PBMC might reduce xenogeneic GvHD (233). Additional administration of purified autologous B cells might ensure efficient autoantibody production (233).

Another important issue is the rapid disappearance of myeloid antigen presenting cells after engraftment of PBMC into immunodeficient mice (29). Since almost all transferred T lymphocytes express HLA-DR after activation, it is conceivable that activated T cells could assume the role of APCs in humanized models (134). However, additional supply of survival factors or repeated administration of myeloid antigen presenting cells such as DCs pulsed with autoantigen might improve the propagation of autoantigen-specific T cell responses. Removal of Treg cells from the human cell graft might further facilitate the establishment of autoimmune responses in humanized mice (292).

A variety of potential RA-associated autoantigens have been described and T cell responses directed against them are extremely heterogeneous among different RA patients, requiring personalized models of RA. These can be easily established in immunodeficient mice by engrafting RA patient-derived PBMCs or SFMCs, which contain autoreactive T cells and are easier to obtain than patient-derived HSCs. Pathogenic T cell clones may be isolated from peripheral blood or synovial fluid of RA patients and expanded *ex vivo* before transferring them into mice. Eventually, it might be necessary to trigger arthritis by activation of human lymphocytes before transfer into mice or by co-administration of arthritogenic antigen.

## Concluding Remarks

Since conventional mouse models of RA are only partially suited to preclinical testing of cell-based therapies, translational research requires a humanized mouse model that accurately mirrors autoimmune processes of human RA and permits its modulation by the transfer of human immunoregulatory cells. Various approaches have been followed during the past decades to establish a humanized model of RA, with only partial success and poor reproducibility. Valuable lessons learnt from translational research models of other autoimmune diseases might help to improve current attempts. A combination of transgenic expression of RA risk alleles and the engraftment of RA patient-derived immune cells and/or RA synovial tissue seems a promising strategy to avoid GvHD and establish chronic autoimmune responses. Improved humanized mouse models of RA would provide a powerful tool for preclinical evaluation of cell-based immunotherapies.

## Author Contributions

The manuscript has been jointly written by KS and JA, and was critically revised by RT, CR, and LS.

### Conflict of Interest Statement

The authors declare that the research was conducted in the absence of any commercial or financial relationships that could be construed as a potential conflict of interest.

## Abbreviations

AA: adjuvant-induced arthritis
ACPAs: anti-citrullinated protein/peptide antibodies
AIA: antigen-induced arthritis
BLT: bone marrow-liver-thymus transplant
BRG: BALB/c-*Rag2*^*null*^ *IL2rg*^*null*^
CII: type II collagen
CFA: complete Freund's adjuvant
CIA: collagen-induced arthritis
CTLA-4: cytotoxic T-lymphocyte-associated protein 4
FOXP3: forkhead-box-protein P3
gp: glycoprotein
GvHD: graft vs. host disease
HLA: human leukocyte antigen
HSCs: hematopoietic stem and progenitor cells
Ig: immunoglobulin
IL: interleukin
IFN: interferon
MHC: major histocompatibility complex
MMC: mutated mouse collagen
NK: natural killer
NOD: non-obese diabetic
NOG: NOD-SCID *Il2rg*^*tm*1*Sug*^
NRG: NOD-*Rag1*^*null*^ *Il2rg*^*null*^
NSG: NOD-SCID *Il2rg*^*tm*1*Wjl*^
PAD: peptidylarginine deiminase
PBMCs: peripheral blood mononuclear cells
PG: proteoglycan
PGIA: Proteoglycan-induced arthritis
RA: rheumatoid arthritis
Rag1/2: recombination-activating genes 1/2
SCID: severe combined immunodeficiency
SE: shared epitope
SFMCs: synovial fluid mononuclear cells
T1D: type 1 diabetes
TCR: T cell receptor
Tg: transgenic
Th: T helper
TNF: tumor necrosis factor
tolDC: tolerogenic dendritic cell
Treg: regulatory T cell.
